# Modeling time delay in the NFκB signaling pathway following low dose IL-1 stimulation

**DOI:** 10.1186/1687-4153-2011-3

**Published:** 2011-06-17

**Authors:** Johannes Witt, Sandra Barisic, Oliver Sawodny, Michael Ederer, Dagmar Kulms, Thomas Sauter

**Affiliations:** 1 Institute for System Dynamics, University of Stuttgart, Pfaffenwaldring 9, 70569 Stuttgart, Germany; 2 Institute of Cell Biology and Immunology, University of Stuttgart, Allmandring 31, 70569 Stuttgart, Germany; 3 Life Sciences Research Unit, University of Luxembourg, 162a, Avenue de la Faïencerie, 1511 Luxembourg, Luxembourg

**Keywords:** IKKbeta, TRAF6, mathematical model, IL-1, UVB

## Abstract

Stimulation of human epithelial cells with IL-1 (10 ng/ml) + UVB radiation results in sustained NFκB activation caused by continuous IKKβ phosphorylation. We have recently published a strictly reduced ordinary differential equation model elucidating the involved mechanisms. Here, we compare model extensions for low IL-1 doses (0.5 ng/ml), where delayed IKKβ phosphorylation is observed. The extended model including a positive regulatory element, most likely auto-ubiquitination of TRAF6, reproduces the observed experimental data most convincingly. The extension is shown to be consistent with the original model and contains very sensitive processes which may serve as potential intervention targets.

## Introduction

The transcription factor NFκB is of central importance in inflammation and anti-apoptotic signaling. Upon stimulation of human epithelial cells with IL-1, NFκB becomes activated due to proteasomal degradation of its cellular inhibitor IκBα. This process requires phosphorylation of IκBα by the upstream kinase IKKβ. Since sustained NFκB-dependent expression of anti-apoptotic genes contributes to the maintenance of a range of cancers, its activity is tightly regulated and terminated by a negative feedback loop, as NFκB promotes IκBα synthesis. Accordingly, various approaches to anti-cancer strategies involve inhibition of the NFκB signaling pathway [1].

Interestingly, NFκB is converted into a pro-apoptotic factor upon stimulation with IL-1 + UVB. The persistence of this effect is ensured by sustained NFκB activity [2] caused by sustained phosphorylation of IKKβ resulting in instant phosphorylation and proteasomal degradation of newly synthesized IκBα. Chronic IKKβ phosphorylation, in turn, is due to UVB-induced inhibition of the responsible phosphatase PP2Ac [2]. We investigated the details of these processes using a systems biological approach, leading to the following ordinary differential equation model of IKKβ phosphorylation and dephosphorylation [3]:(1)

with [ILR](0) = 1, [ILRc](0) = 0, [IKKp](0) = 0, [PP2A](0) = 1, [IKK] = 1 - [IKKp]. By western blot analysis, we measure IKKp_obs = IKKp scale_IKK. The factor scale_IKK describes the unknown ratio between the strength of the IKKp band on the gel and the concentration of IKKp.

The system variables describe the normalized concentrations of IL-1 receptor [ILR], IL-1 receptor complex [ILRc], phosphorylated and unphosphorylated IKKβ ([IKKp] and [IKK]), and PP2Ac [PP2A], the inputs il(t) and uv(t) describe IL-1 concentration and UVB radiation. Due to the normalization, all kinetic parameters of this model are given in s^-1 ^except for *k*_a _(nM^-1 ^s^-1^), since il(t) is given in nM.

In the original model [3] the effects of the signaling cascade are considered to be negligible, so that the IKKβ kinase [kin] is assumed to have the same time course as [ILRc], i.e., [kin] = [ILRc]. Despite this simplifying assumption the model works well for high IL-1 doses (10 ng/ml, corresponding to 0.588 nM), indicating that the simplification is justified in this dose range.

**Figure 1:**
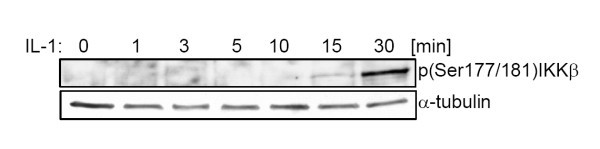
**IKKβ phosphorylation is delayed following stimulation with 0.5 ng/ml IL-1 (adapted from **[3]).

While IKKβ is very rapidly phosphorylated upon 10 ng/ml IL-1 stimulation, delayed IKKβ phosphorylation could be observed upon 0.5 ng/ml or 0.029 nM IL-1 stimulation (Figure 1). This slowly increasing phosphorylation activity is only insufficiently reproduced by the original model, so that a more detailed model of the signaling cascade appears appropriate for low IL-1 doses. However, the mechanism causing the signal delay is unknown to date. In principle, various reasons for signal delay are conceivable. Among the most prominent examples are double phosphorylation as occurring, e.g., in the MAPK cascade [4], mechanisms with irreversible inhibitors and positive feedback mechanisms. Here we will investigate these three mechanisms by extending the original model by each of them separately to predict the most probable explanation for the delay.

## Modeling

**Figure 2:**
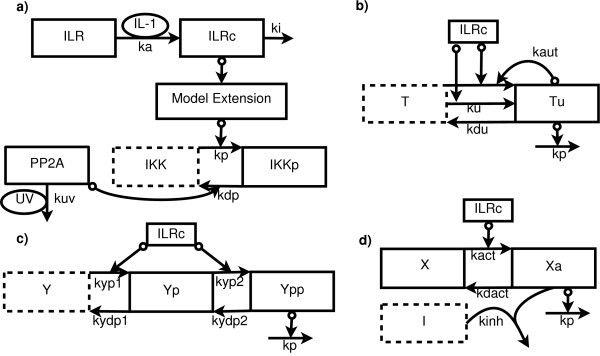
**Graphical representation of the four model variants**. Schematic representation of the original IKKβ phosphorylation model **(a) **with the extensions positive feedback/TRAF **(b)**, double phosphorylation **(c)**, and irreversible inhibitor **(d)**. Ellipses denote model inputs, boxes with solid lines denote state variables, boxes with dotted lines denote species calculated from mass conservation relations. Normal arrows denote mass flows, circles at an arrow tail signify that the reactant participates catalytically in the reaction.

Each of the three potential mechanisms delaying the signaling cascade following low doses of IL-1 is modeled as a separate building block (see Figure 2). It is then used to calculate [kin] as a function of [ILRc], where [kin] is [Tu] or [Ypp] or [Xa], depending on the potential mechanism (see below).

The positive feedback mechanism describes the autocatalyzed activation of a protein T to the activated form Tu. This mechanism is given as(2)

with [Tu](0) = 0 and [*T*] = 1 - [Tu].

In the double phosphorylation mechanism, the active form Ypp of a protein Y is reached after two sequential modifications (e.g., phosphorylations) catalyzed by ILRc:(3)

with [Y] = 1 - [Yp] - [Ypp], [Yp](0) = 0, [Ypp](0) = 0. In the irreversible inhibitor mechanism, an irreversible inhibitor I blocks the activated form Xa of some protein X within the signaling cascade. The mechanism is implemented as(4)

where [X](0) = 1, [Xa](0) = 0, [I](0) = I_0_. Note that [I] can also be obtained from the mass conservation relation [I] = [X] + [Xa] + I_0 _- 1. All concentrations except [I] are scaled without loss of generality so that all non-zero initial concentrations except I_0 _are 1 (see the analogous argumentation in Additional File 1 of [3]). The exception of [I] is due to the term *k*_inh _[Xa][I] which occurs in the differential equations for both  and  Figure 2 shows a schematic representation of the original model and the three extensions.

While no specific irreversible inhibitor mechanism is known within the IL-1 signaling cascade, the double phosphorylation mechanism might, e.g., correspond to IRAK-1 or IRAK-4 phosphorylation [5]. A promising candidate for the positive feedback mechanism in the IL-1 signaling cascade is TRAF6: The E3-ligase TRAF6 is a key mediator of IL-1 induced signaling because its auto-ubiquitination is required to activate IKKβ [6]. Hence, the positive feedback mechanism might describe TRAF6 auto-ubiquitination. In the following, we therefore refer to this model extension as the TRAF extension. Note that, while no other comparable mechanism is known to date within the IL-1 signalling cascade, the TRAF module might also represent a different, yet unknown positively auto-regulated mechanism within the signalling cascade.

Experimental data for 0.5 ng/ml IL-1 stimulation were obtained from three western blot experiments measuring IKKβ Ser177/181 phosphorylation in human epithelial carcinoma cell line KB following IL-1 stimulation with and without UVB (300 J/m^2^) costimulation until 120 min post-stimulation as described in [3]. They were subsequently extended with data from two western blots covering the first 30 min after IL-1 stimulation without UVB. Data for the 10 ng/ml IL-1 ± UVB stimulation are taken from [3]. Maximal IKKβ phosphorylation was clearly lower for low (0.5 ng/ml) than for high (10 ng/ml) IL-1 stimulation, so that the maximal value could not be used as a reference value for scaling. Therefore, a separate scaling factor was used for IKKβ following 0.5 and 10 ng/ml IL-1 stimulation.

Modeling and analysis was performed with the Matlab (The MathWorks) based software tool PottersWheel (http://www.potterswheel.de freely available for academic use) [7], analogously to the procedures described in [3]. Particularly, the χ^2 ^value was used as objective function, with

where *M *is the number of stimulations (i.e., *M *= 4), *N*_*i *_is the number of data points under stimulation *i*, *y*_*ij *_is data point *j *under stimulation *i *with standard deviation σ_*ij *_and *y*(*t*_*ij*_, θ) is the simulated value at time point *j *under stimulation *i *for the parameter vector θ.

The upper bound of the scaling factor for low IL-1 stimulations was set to 4, as obtained from experimental data (not shown). Upper bounds for the remaining parameter values were derived as described in [3], lower bounds were not required.

## Results and Discussion

### Comparison of the different model extensions

Fitting of the model with its different extensions to the experimental data yields better χ^2 ^values than the original model for all extensions (Table 1). Comparison of the parameter values with the parameter values of the original model showed that the parameters downstream of the model extension, i.e., *k*_uv_, *k*_p, _and *k*_dp_, remain basically unchanged (Table 2).

**Table 1:** χ^2 ^values and AICc of the model and its extensions fitted to the four experimental data sets

Model	χ^2 ^value	AICc
Original (a)	45.8	124.9
TRAF (b)	23.1	113.7
Double phosphorylation (c)	28.3	123.5
Irreversible inhibitor (d)	27.0	122.2

**Table 2:** Values of the kinetic parameters downstream of the model extension in the original model [3] and in the extended models

Model	*k*_p _(s^-1^)	*k*_dp _(s^-1^)	*k*_uv _(s^-1^)
Original (a)	0.095	7.6e-4	2.4e-4
TRAF (b)	0.095	9.3e-4	2.6e-4
Double phos. (c)	0.095	7.5e-4	2.3e-4
Irr. inhibitor (d)	0.095	7.2e-4	2.3e-4

**Figure 3:**
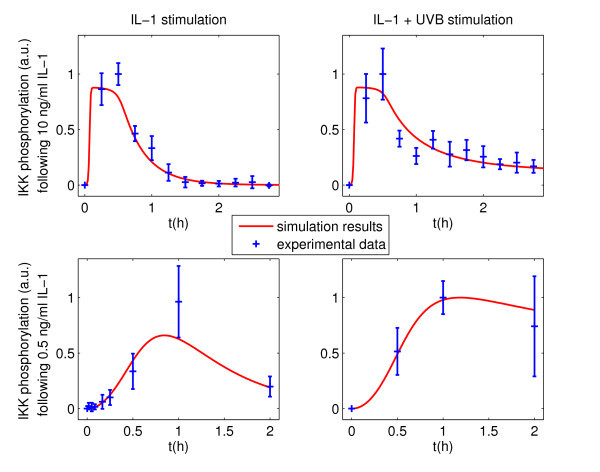
**IKKβ phosphorylation following different stimulations - experimental data and simulation results of the model with TRAF extension**. Columns show stimulation with IL-1 or IL-1 + UVB (300 J/m^2^) radiation, respectively, applied at *t *= 0 s. Rows indicate whether a high (10 ng/ml) or low (0.5 ng/ml) IL-1 dose was applied. A different scaling factor is chosen for the data for 10 and 0.5 ng/ml IL-1 stimulation.

While all model extensions are able to reproduce the delay (Figure 3; Figures S1 and S2 in Additional file 1), the TRAF extension is the model structure with the least degrees of freedom and lowest χ^2 ^value. Consequently, it also yields the best (lowest) value of the Corrected Akaike Information Criterion, AICc, which compares the goodness-of-fit of models with a different number of parameters (Table 1). In contrast, the other extensions only perform about as well as the original model in terms of AICc.

The TRAF extension convincingly reproduces the experimental data and especially the signal delay (Figure 3), and a biological counterpart to this mechanism exists within the IL-1 signaling cascade. In the following, we therefore focus on the TRAF extension. All parameter values of this extension are given in Table S1 in Additional file 1.

### Detailed analysis of the TRAF6 model extension

Fixing of all model parameters also occurring in the original model to their value reported in [3] yields only slightly worse fit quality of χ^2 ^= 24.3. Remarkably, the fit quality is still better than the fit quality of the original model [3] (χ^2 ^= 24.5), where only the data sets for 10 ng/ml IL-1 doses ±UVB are considered. As to the time course, the most prominent difference of the model extension compared to the original model consists in a longer plateau phase of the maximum concentrations, which is in better agreement to the measured data. In summary, the model extension is consistent to the model presented in [3] and justifies its additional complexity with a considerable enhancement of the fit quality and a consistent qualitative behavior for low doses.

Although the parameter fixing performed above suggests that identifiability in the extended model might be worse than in the original one, identifiability analysis shows that all parameter values are identifiable from the given data: 2000 fits were performed with initial parameter values obtained by randomly perturbing the best parameter values by up to four orders of magnitude. The standard deviation of the parameter sets of the best 800 fits is not more than 1% for each parameter.

**Figure 4:**
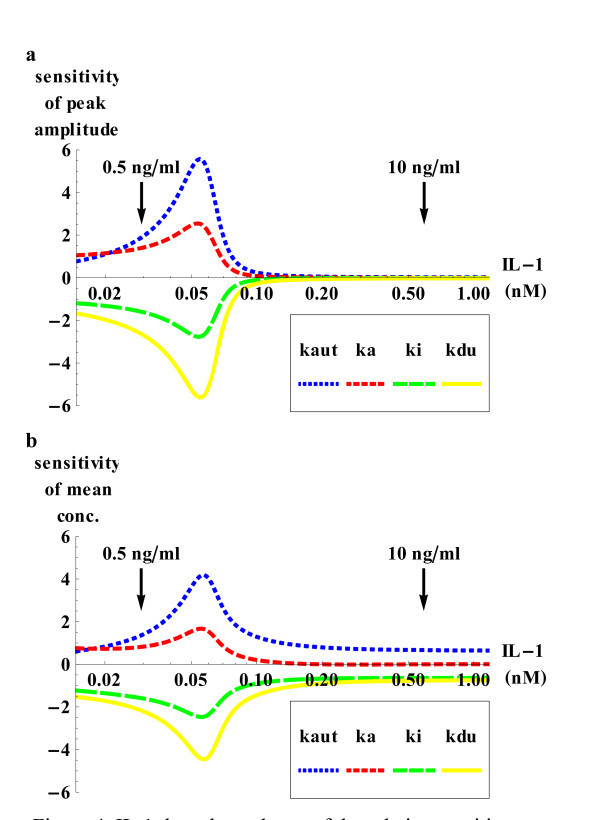
**Sensitivity analysis**. The relative sensitivity of the **(a) **peak and **(b) **mean IKKp concentration to perturbations in the parameters *k*_a_, *k*_*i*_, *k*_aut_, and *k*_du _is highly dependent on the IL-1 dose. Sensitivities were calculated and plotted using *Mathematica *8.0.

Furthermore, we calculated the relative sensitivity

of *x *against perturbations in parameter *p*_*i *_following IL-1 stimulation for two time-independent characteristics *x *of IKKp(*t*): the IKKp peak amplitude, *x *= max(IKKp(*t*)) and the mean of IKKp within the longest observed time period,

For both sensitivity measures, a high dose dependency can be observed (Figure 4): for 10 ng/ml (0.588 nM) IL-1, parameter perturbations result in relatively weaker changes of the mean IKKp value (|*s*_*i*_| < 1), and have almost no effect on the peak amplitude. In contrast, the system is quite sensitive upon 0.5 ng/ml (0.029 nM) IL-1 stimulation: for several parameters, perturbations have an amplified effect on both peak amplitude and average IKKp value (|*s*_*i*_| > 1). Furthermore, the system is sensitive over a wide dose range, with sensitivities of up to ±5 (Figure 4).

Taken together, the positive feedback regulation presumably mediated via TRAF6 auto-ubiquitination [6] represents a consistent, well identifiable extension of the IKKβ phosphorylation model that reproduces the time delay at low IL-1 stimulations and is in accordance with literature findings.

On the other hand, it should be noted that determination of low levels of phosphorylated IKKβ is an experimentally challenging task, so that the values for 0.5 ng/ml IL-1 stimulation should be regarded as somewhat semiquantitative measurements despite the partially low standard deviation. Against this background, though the postulated model consistently explains the experimental data one should neither completely reject the other two investigated mechanisms, which also perform well, nor lose sight of other potential delay mechanisms. Nevertheless, our results may provide an indication that the reported TRAF6 auto-ubiquitination [6] can indeed be interpreted as a positive feedback loop.

The very good accordance of the relatively small model with the experimental data together with the reported findings [6] make TRAF6 auto-ubiquitination a promising candidate for further research. Several system parameters could be characterized as very sensitive at low IL-1 doses. The respective processes therefore qualify as potential intervention targets in cancer therapy. The systems biological approach in combination with the necessary experimental validation can help to further elucidate important molecular mechanisms.

## Abbreviations

NFκB: nuclear factor κB; IL-1: interleukin-1; IκBα: inhibitor of κBα; IKKβ: inhibitor of κB kinase: subunit β; UVB: ultraviolet-B radiation; PP2Ac: protein phosphatase 2A: subunit c; MAPK: mitogen-activated protein kinase; IRAK1/4: interleukin-1 receptor-associated kinase 1/4; TRAF6: TNF receptor associated factor 6.

## Competing interests

The authors declare that they have no competing interests.

## Supplementary Material

Additional file 1 **Figure S1. IKKβ phosphorylation following different stimulations - experimental data and simulation results of the model with buffer extension**. Columns show stimulation with IL-1 or IL-1 + UVB (300 J/m^2^) radiation, respectively, applied at *t *= 0 h. Rows indicate whether a high (10 ng/ml) or low (0.5 ng/ml) IL-1 dose was applied. A different scaling factor is chosen for the data for 10 and 0.5 ng/ml IL-1 stimulation. **Figure S2**. IKKβ phosphorylation following different stimulations - experimental data and simulation results of the model with double phosphorylation extension. Columns show stimulation with IL-1 or IL-1 + UVB (300 J/m^2^) radiation, respectively, applied at *t *= 0 h. Rows indicate whether a high (10 ng/ml) or low (0.5 ng/ml) IL-1 dose was applied. A different scaling factor is chosen for the data for 10 and 0.5 ng/ml IL-1 stimulation. **Table S1**. Parameter values of the model with TRAF extension Supplemental table Click here for file
