# Supplementary material for: Modeling time delay in the NFκB signaling pathway following low dose IL-1 stimulation
Source: EURASIP J Bioinform Syst Biol. 2011 Jun 17;2011(1):3. doi: 10.1186/1687-4153-2011-3 (PMC3171178; doi:10.1186/1687-4153-2011-3)
Supplement: Additional file 1 — Figure S1. IKKβ phosphorylation following different stimulations - experimental data and simulation results of the model with buffer extension. Columns show stimulation with IL-1 or IL-1 + UVB (300 J/m2) radiation, respectively, applied at t = 0 h. Rows indicate whether a high (10 ng/ml) or low (0.5 ng/ml) IL-1 dose was applied. A different scaling factor is chosen for the data for 10 and 0.5 ng/ml IL-1 stimulation. Figure S2. IKKβ phosphorylation following different stimulations - experimental data and simulation results of the model with double phosphorylation extension. Columns show stimulation with IL-1 or IL-1 + UVB (300 J/m2) radiation, respectively, applied at t = 0 h. Rows indicate whether a high (10 ng/ml) or low (0.5 ng/ml) IL-1 dose was applied. A different scaling factor is chosen for the data for 10 and 0.5 ng/ml IL-1 stimulation. Table S1. Parameter values of the model with TRAF extension Supplemental table [file 1687-4153-2011-3-S1.PDF]

Supplementary Table 1. Parameter values of the model with TRAF extension.

Parameter	value	dimension
ka	0.014	$nM^{-1}\ s^{-1}$
ki	5.6e-4	s^{-1}
kp	0.095	s^{-1}
kdp	9.3e-4	s^{-1}
kuv	2.6e-4	s^{-1}
kaut	0.095	s^{-1}
ku	9.3e-5	s^{-1}
kdu	0.041	s^{-1}
scaleIKK_high	0.90	-
scaleIKK_low	4.0	-

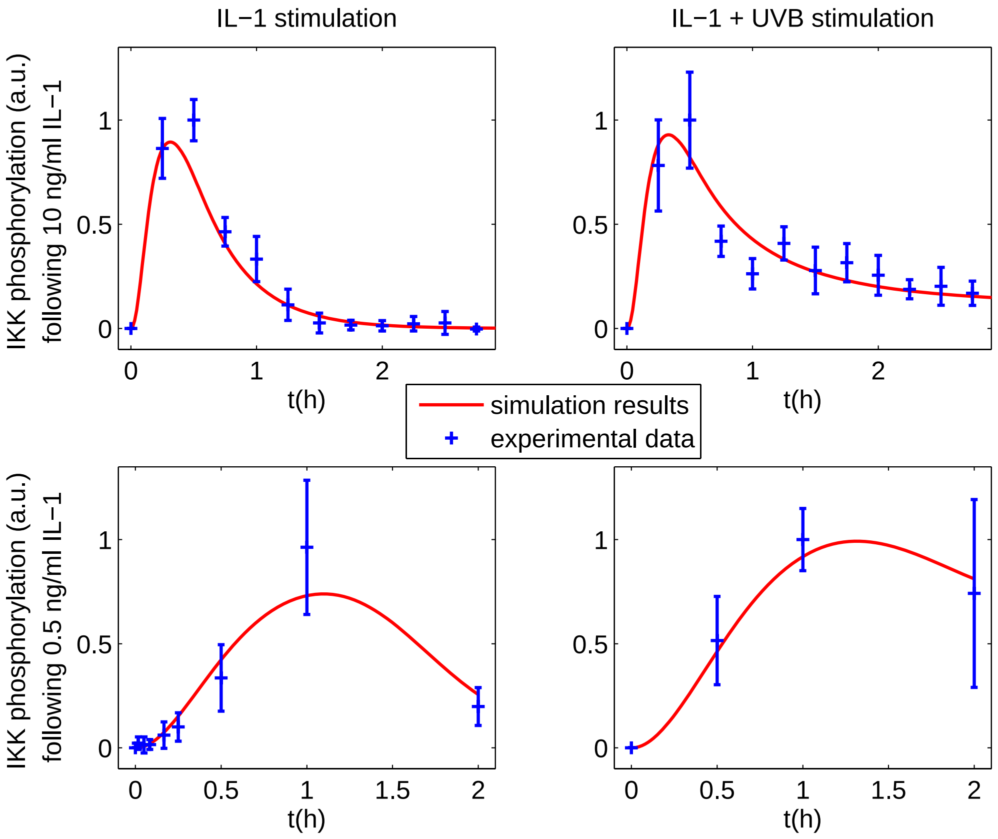

Supplementary Figure 1. IKKβ phosphorylation following different stimulations – experimental data and simulation results of the model with buffer extension. Columns show stimulation with IL-1 or IL-1+UVB (300 J/m²) radiation, respectively, applied at t = 0 h. Rows indicate whether a high (10 ng/ml) or low (0.5 ng/ml) IL-1 dose was applied. A different scaling factor is chosen for the data for 10 ng/ml and 0.5 ng/ml IL-1 stimulation.

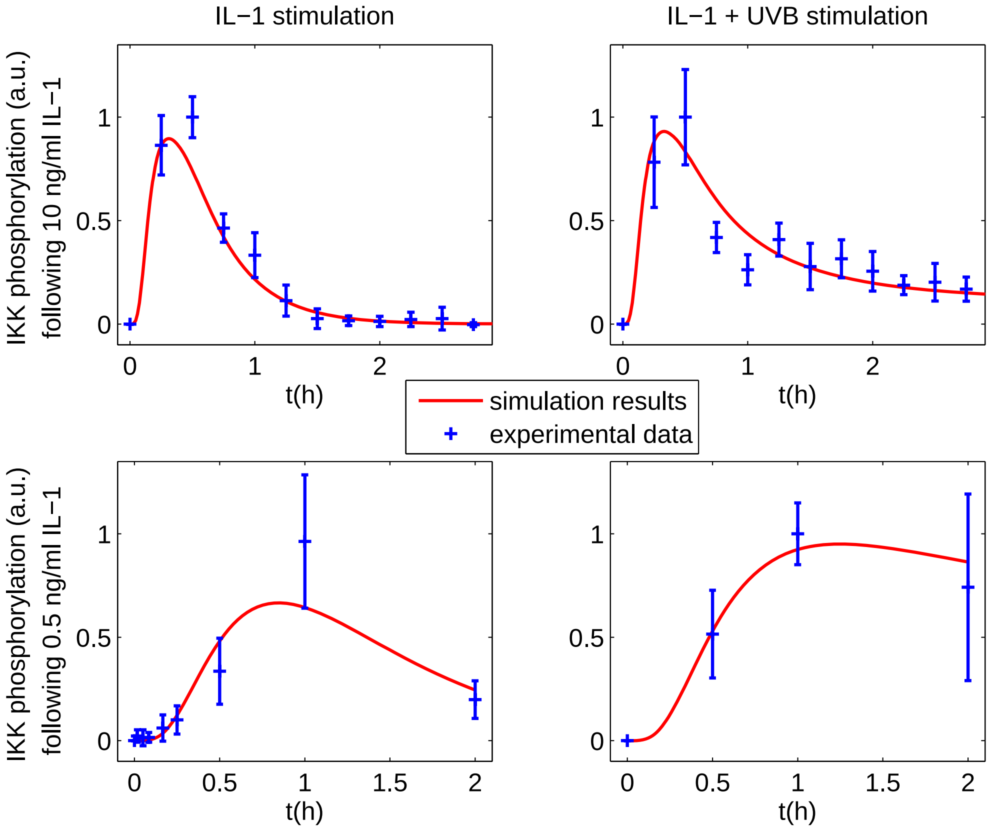

Supplementary Figure 2. IKKβ phosphorylation following different stimulations – experimental data and simulation results of the model with double phosphorylation extension. Columns show stimulation with IL-1 or IL-1+UVB (300 J/m²) radiation, respectively, applied at t = 0 h. Rows indicate whether a high (10 ng/ml) or low (0.5 ng/ml) IL-1 dose was applied. A different scaling factor is chosen for the data for 10 ng/ml and 0.5 ng/ml IL-1 stimulation.
